# Hemodynamics and Wall Shear Stress of Blood Vessels in Aortic Coarctation with Computational Fluid Dynamics Simulation

**DOI:** 10.3390/molecules27041403

**Published:** 2022-02-18

**Authors:** Gi-Beum Kim, Kwang-Hyun Park, Seong-Jong Kim

**Affiliations:** 1 Eouidang Agricultural Company, 4086-4 Chunhang-ro, Sanggwan-myeon, Wanju-gun 55360, Korea; 2 Department of Emergency Medicine and BioMedical Science Graduate Program (BMSGP), Chonnam National University, Gwangju 61469, Korea; khpark@jbnu.ac.kr; 3 School of Chemical Engineering, College of Engineering, Jeonbuk National University, Jeonju 54896, Korea

**Keywords:** abdominal aortic aneurysm, computational fluid dynamics (CFD), atherosclerosis, vortex flow, wall shear stress, wall load

## Abstract

The purpose of this study was to identify the characteristics of blood flow in aortic coarctation based on stenotic shape structure, stenosis rate, and the distribution of the wall load delivered into the blood vessels and to predict the impact on aneurysm formation and rupture of blood vessels by using a computational fluid dynamics modeling method. It was applied on the blood flow in abdominal aortic blood vessels in which stenosis occurred by using the commercial finite element software ADINA on fluid-solid interactions. The results of modeling, with an increasing stenosis rate and Reynolds number, showed the pressure drop was increased and the velocity was greatly changed. When the stenosis rate was the same, the pressure drop and the velocity change were larger in the stenosis with a symmetric structure than in the stenosis with an asymmetric one. Maximal changes in wall shear stress were observed in the area before stenosis and minimal changes were shown in stenosis areas. The minimal shear stress occurred at different locations depending on the stenosis shape models. With an increasing stenosis rate and Reynolds number, the maximal wall shear stress was increased and the minimal wall shear stress was decreased. Through such studies, it is thought that the characteristics of blood flow in the abdominal aorta where a stenosis is formed will be helpful in understanding the mechanism of growth of atherosclerosis and the occurrence and rupture of the abdominal aortic flow.

## 1. Introduction

The abdominal aorta is the major blood vessel of the body that runs and carries blood from the heart to the chest, stomach, intestine, kidneys, and down to the abdomen and the rest of the body. An aneurysm is an abnormal dilatation of blood vessel that involves all three layers of the vessel wall (intima, media, and adventitia). An aortic aneurysm is such a dilatation of the aorta, and the most common form of aneurysm is the abdominal aortic aneurysm (AAA). Most occur infra-renally (below the kidneys) [1]. When the diameter of the abdominal aorta exceeds 3 cm it is considered to be an aneurysm. Identifying an aortic aneurysm is important because it may progress and cause blockage of the blood supply to various organs (i.e., kidney, intestines, etc.) and may contain a thrombus that might embolize to distal organs or may burst and cause severe, often life-threatening hemorrhage and death [2]. Approximately 500,000 AAAs are diagnosed worldwide each year, resulting in 15,000 deaths per year in the USA alone [3].

Causes of the development of AAA include stenosis, atherosclerosis, infection, trauma, arteritis, cystic medial necrosis, hypertension, diabetes, and smoking. Stenosis is the abnormal narrowing of a blood vessel, sometimes called a stricture. Aortic sclerosis, the precursor of aortic stenosis (AS), has been found in approximately one-third of patients over the age of 65 years [4,5]. In most patients, the underlying cause is calcific AS [6]. This is a chronic, progressive disease that begins with the thickening and calcification of the valve cusps without hemodynamic significance and ends in heavily calcified, stiff cusps that cause severe valve stenosis. Recent studies have shown that this is not only a degenerative process due to mechanical stress but also an active process, involving inflammation and lipid infiltration, similar to that seen in atherosclerosis [4,5,6,7]. Aortic stenosis (AS), also known as aortic valve stenosis, is classified as valvular heart disease. The increasing prevalence of combined severe aortic stenosis and AAA with the aging of the population indicates AS and AAA share some various risk factors, such as coarctation of the aorta, midaortic dysplastic syndrome, atherosclerosis, aortic dissection, or various intra/periaortic diseases and/or results of aortic surgical repair [8,9]. Epidemiological studies have confirmed that AS and atherosclerosis share several common risk factors: male sex, older age, hypertension, diabetes, smoking, and elevated levels of low-density lipoprotein (LDL) cholesterol and lipoprotein [5,7]. These observations have led to the proposal of pharmacological strategies already used in atherosclerosis, e.g., angiotensin-converting enzyme inhibitors and hydroxymethylglutaryl coenzyme. Reductase inhibitors (statins) may slow the progression of AS. The AAA was long considered to be caused by atherosclerosis because the walls of the AAA are frequently affected heavily. However, this theory cannot be used to explain the initial defect and the development of occlusion, which is observed in the process. Greater than 90% of people who carry an AAA have smoked at some point in their life [6]. The frequency varies strongly between males and females. The peak incidence is among males around 70 years of age; the prevalence among males over 60 years totals 2–6%. The frequency is much higher in smokers than in non-smokers (8:1), and the risk decreases slowly after smoking cessation [5]. Other risk factors include hypertension and male sex. In the USA, the incidence of AAA is 2–4% in the adult population [6]. AAA is 4–6 times more common in male siblings of known patients, with a risk of 20–30% [10].

Abdominal aortic aneurysms (AAAs) may be considered as a particular form of atherothrombosis characterized by high levels of proteolytic activity leading to dilation and eventually to rupture of the aortic wall [11,12,13]. Rupture of the AAA occurs in 1–3% of men aged 65 or more, and mortality is 70–95% [14]. The most common treatment for a large, unruptured aneurysm is open surgical repair by a vascular surgeon. This procedure involves an incision from just below the breastbone to the top of the pubic bone. The surgeon then clamps off the aorta, cuts open the aneurysm, and sews in a graft to act as a bridge for the blood flow. The blood flow then goes through the plastic graft and no longer allows the direct pulsation pressure of the blood to further expand the weak aorta wall [15].

The purpose of this study was to identify the characteristics of blood flow in aortic stenosis on stenotic shape structure, stenosis rate, and the distribution of wall load delivered into the blood vessels and to predict the impact on aneurysm formation and rupture of blood vessels by using a computational fluid dynamics (CFD) modeling method.

## 2. Results and Discussion

**Figure 1:**
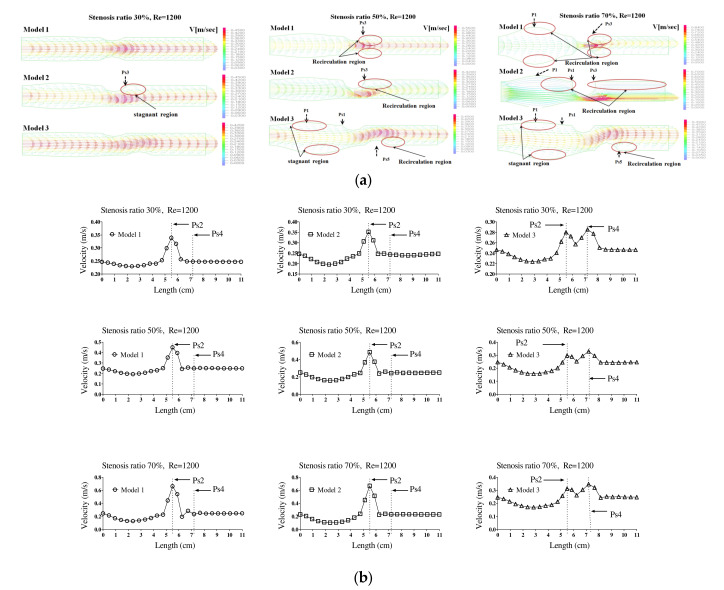
The velocity distribution and average velocity in the continuous flow field based on changes to the stenosis ratio at each point of the modules. (**a**) Velocity distribution, (**b**) average velocity.

Figure 1 shows the velocity distribution and average velocity in the continuous flow field based on changes to the stenosis ratio at each point of the modules. When the boundary condition was assumed to be a fully developed flow in the entrance region, velocity distribution for the axial direction had a complete parabolic form in model 1; it showed the highest flow velocity in the center and the flow velocity close to ‘0’ in the wall. In models 2 and 3, the fluid flow was asymmetric and it was concentrated on one side. Particularly, in model 2, when the stenosis rate was 50%, the flow separation was made at Ps3, in which the stenosis ended. When the stenosis rate was 70%, the flow separation was made at Ps3 and P2. However, in model 1 when the stenosis rate was 50% and in all models when the stenosis rates were 50% and 70%, flow velocity accelerated rapidly in the region of the stenosis and it decreased rapidly in the area after the stenosis, in which the diameter increase again. At the time, the vortex flow occurred and the recirculation region was formed. In addition, the stagnation region was formed, in which the flow velocity was ‘0’ in the wall of the blood vessel in the region ahead of the stenosis. In Models 1 and 2, the highest flow velocity was shown at Ps2. In Model 3, the highest flow velocity was shown at Ps4. Models 1 and 2 showed the highest velocity at Ps2, in which the diameter of blood vessels was minimized. Model 3 showed the highest velocity at Ps4 when the stenosis was more than 50%. When the stenosis was the same, the average velocity in the area of the stenosis was similar to each other in models 1 and 2, but it was relatively low in model 3. In addition, all models showed the tendency of reduction in velocity from the entrance region to the Ps1. In Models 1 and 2, when the stenosis rate was 70% and the Reynolds number was 1200, the maximum velocity at Ps2 was approximately 1.8 times higher than when the stenosis rate was 30%. It was approximately 1.2 times higher than that in model 3.

**Figure 2:**
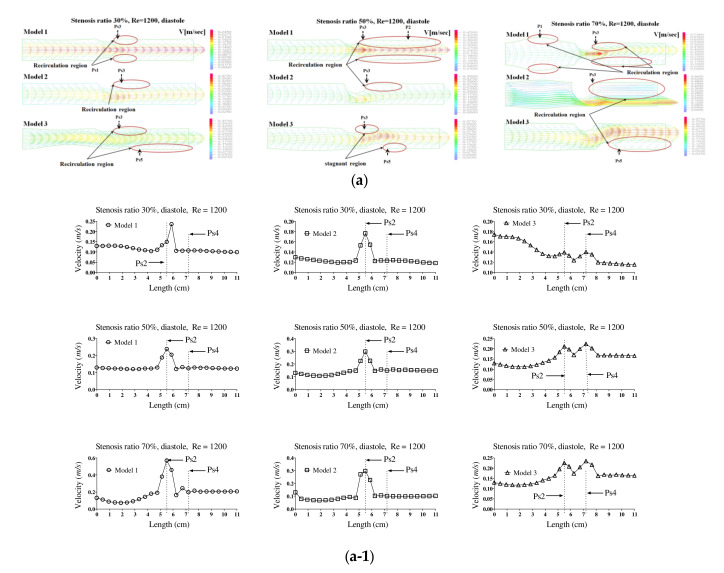

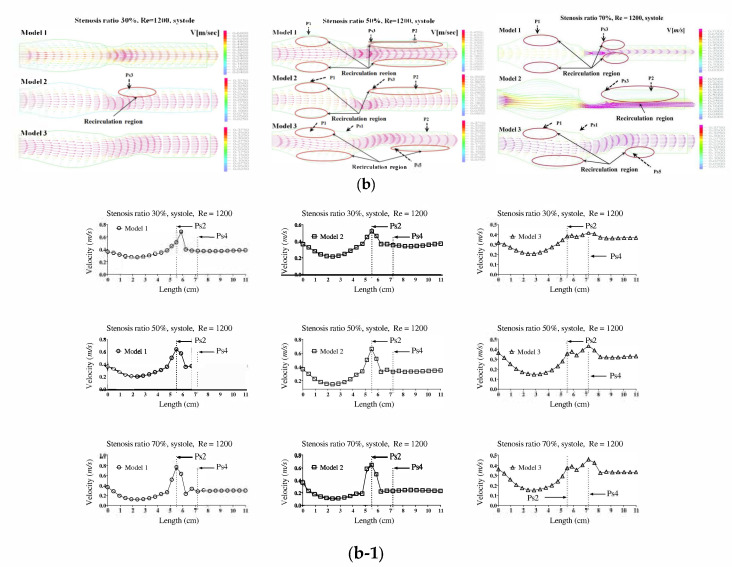
The velocity distribution and average velocity in the pulsatile flow field based on changes to the stenosis ratio at each point of the modules. (**a**) Diastolic velocity distribution and (**a-1**) diastolic average velocity; (**b**) systolic velocity distribution and (**b-1**) systolic average velocity.

Figure 2a,b show the velocity distribution and average velocity in the pulsatile flow field based on changes to the stenosis ratio at each point of the modules. The vortex was partially formed as the stenosis rate increased during diastole, and then the recirculation region and stagnant layer were formed. Especially when the stenosis rate was 70%, the velocity distribution was very complex and a biased flow was shown. The location for the recirculation region and stagnant layer was shown variously based on the shape of the stenosis. When the stenosis rate was 70%, the velocity distribution was biased on one side with increasing the Reynolds number and the recirculation region was formed near the wall of the blood vessel after Ps3 (in Figure 2a). It was found that the recirculation region was formed in models 1 and 2 ahead of the stenosis with increasing the stenosis rate and the Reynolds number. It is thought that this phenomenon was caused by the reverse flow of blood due to the decelerating force reducing the amount of blood flow near the Ps1 in which the diameter of blood vessel was decreased. After Ps3, the vortex rapidly developed as the stenosis rate and Reynolds number increased. Thus, it was found that a bigger recirculation region was formed. However, during systole, the velocity distribution was similar to the results of a continuous flow. In the case of a low stenosis rate, model 1 showed the relatively smooth flow, and models 2 and 3 showed the occurrence of a recirculation region ahead of the Ps1 region. In regions before and after Ps2, flow disturbance such as flow separation and vortex occurred and the asymmetry of the stenosis shape had a big impact on this flow disturbance. In the entire regions, the velocity of blood flow was lowered and the vortex was formed in the wall of the blood vessel. In this phenomenon, the range of occurrence of the vortex was increasing with an increase in the Reynolds number and stenosis rate. In model 3, it was found that the center part of the recirculation flow formed in the region ahead of Ps1 and moved to the entrance region with an increase in the Reynolds number. In models 1 and 2, Ps2 showed the highest velocity during diastole, but the average velocity during systole was similar to the results of the continuous flow. Ps2 showed the highest velocity in Models 1 and 2, and Ps4 showed the highest velocity in model 3. The average velocity decreased linearly from the entrance region to Ps1, but it tended to be increased after that. The lowest velocity was shown at P1. With the same stenosis rate, it was found that this tendency became worse as the Reynolds number increased.

**Figure 3:**
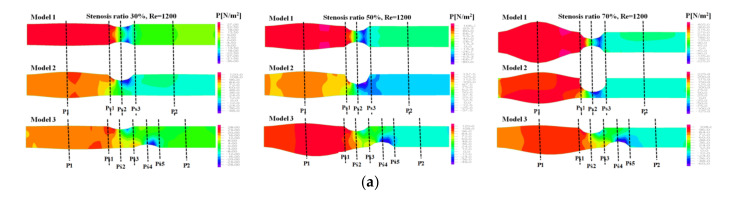

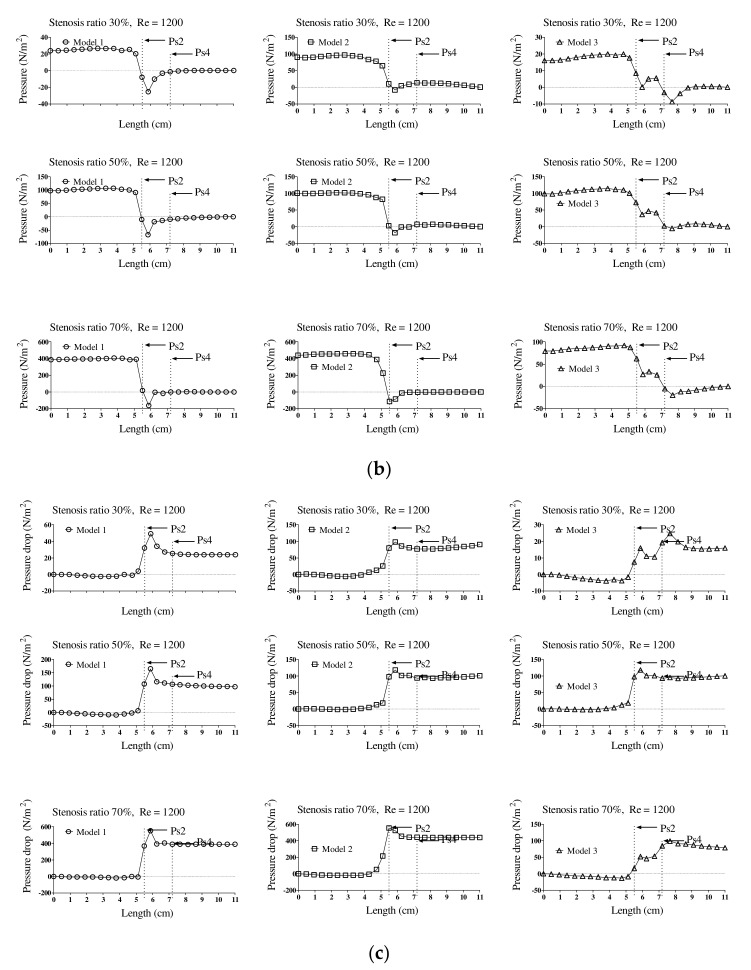
The pressure distribution, the average pressure, and pressure drop in the continuous flow field based on changes to the stenosis ratio at each point of the modules. (**a**) Pressure distribution, (**b**) average pressure, (**c**) pressure drop.

Figure 3 shows the pressure distribution, the average pressure, and pressure drop in the continuous flow field based on changes to the stenosis ratio at each point of the module. The average pressure increased linearly from the entrance region to Ps1, but it rapidly decreased after Ps1. A minimum value was shown in Ps2, but the pressure was not changed after Ps3. In Model 1, when the stenosis rate was 70% and Reynolds number was 1200, the maximum velocity was approximately 13 times higher than when the stenosis rate was 30%. It was approximately 5.5 times higher than that in model 2. It was approximately 4.5 times higher than that in model 3. In the case of model 1 with symmetric stenosis, as the stenosis rate increased, the maximum pressure was shown in the region ahead of Ps1. As the Reynolds number and stenosis rate increased, a significant change in pressure was shown.

**Figure 4:**
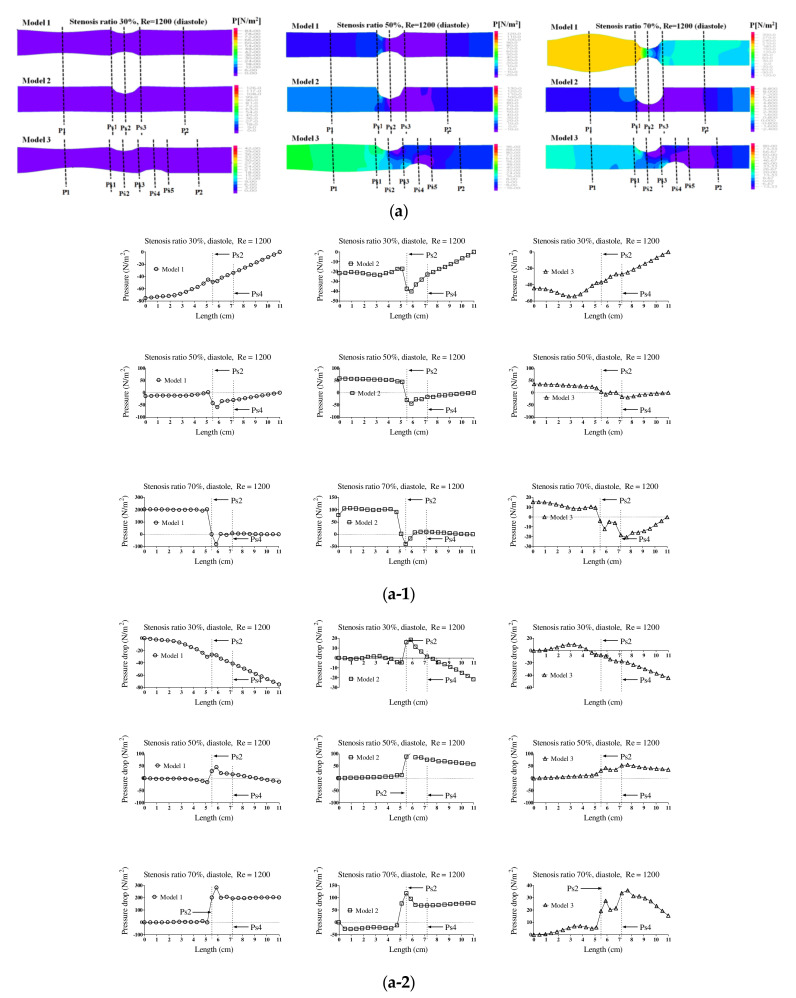

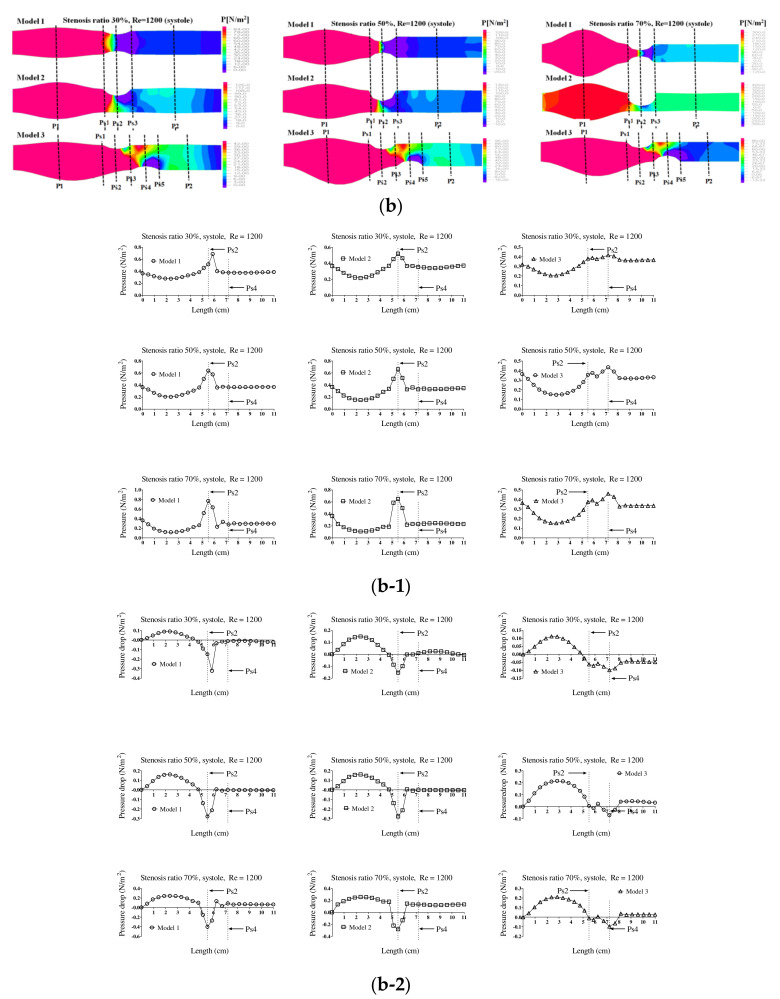
The pressure distribution, the average pressure, and pressure drop in the pulsatile flow field based on changes to the stenosis ratio at each point of the modules. Diastolic (**a**) pressure distribution, (**a-1**) average pressure, and (**a-2**) pressure drop; systolic (**b**) pressure distribution, (**b-1**) average pressure, and (**b-2**) pressure drop.

Figure 4 shows the pressure distribution, the average pressure, and pressure drop in the pulsatile flow field based on changes to the stenosis ratio at each point of the modules. When the stenosis rate was 30% during diastole, the average pressure was the lowest in the entrance region and it was the highest in the exit region. In the case of a low stenosis rate, the average pressure generally had a negative value. High pressure was shown in the region ahead of Ps1 with an increasing stenosis rate and Reynolds number. When the stenosis rate was more than 70%, it was found that the pressure drop was increasing with an increasing Reynolds number. The average pressure during systole was similar to the result of a continuous flow and its value was relatively high. Changes in pressure in the blood vessel were gradually increasing until it reached the Ps1, and it rapidly decreased at Ps1. The lowest value was shown at Ps2 in models 1 and 2 and it was shown at Ps4 in model 3. The maximum pressure was shown at P1 in models 1 and 2 and it was shown at Ps1 in model 3. In the case of a low stenosis rate, the pressure tended to be increasing after Ps3 and then decreasing. When the stenosis rate was more than 50%, the pressure distribution was significantly changed in the region ahead of Ps1 with an increasing Reynolds number during diastole. For the pressure distribution during systole, it was found that a significantly high pressure was shown in all regions of the stenosis and the minimum pressure was shown in the stenosis region.

**Figure 5:**
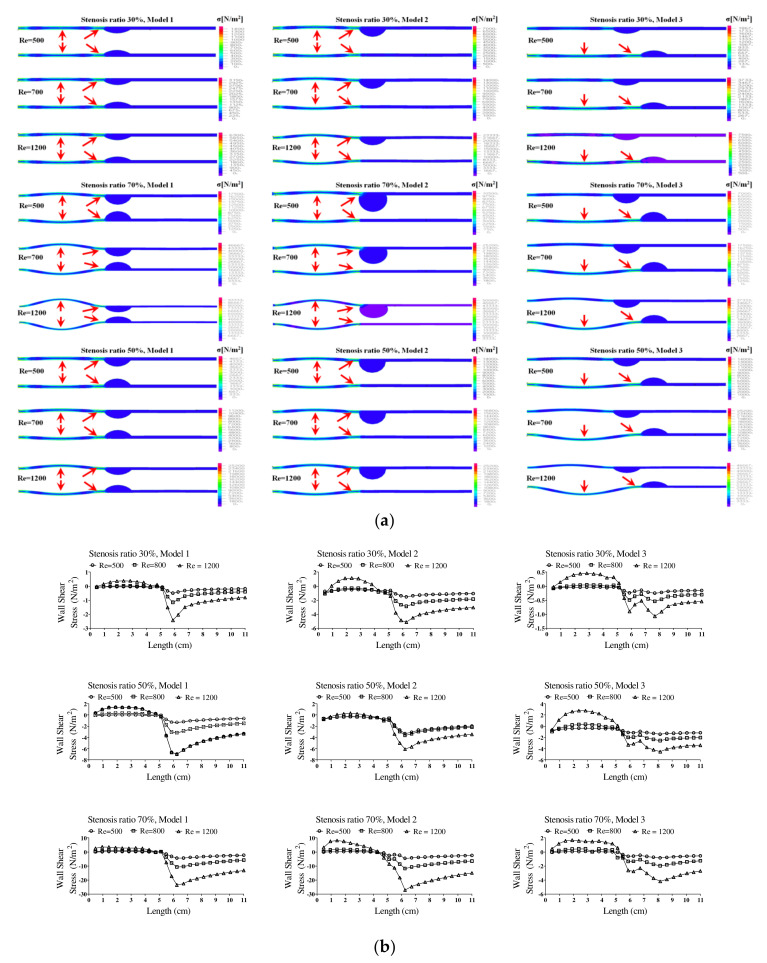
The wall load distribution and wall shear stress on the wall of blood vessels in the continuous flow. (**a**) Wall load distribution, (**b**) wall shear stress.

Figure 5 shows the wall load distribution and wall shear stress (WSS) on the wall of the blood vessels in the continuous flow field based on changes to the stenosis ratio at each point of the modules. Such WSS is closely associated with growth of the stenosis part in the blood vessel and rupture of the blood vessel. In addition, it is known that intimal thickening occurs easily in an area with a low WSS. Such intimal thickening progresses as the initial atherosclerotic plaque [16]. The WSS rapidly decreased at Ps1 and it gradually increased at Ps3. A rapidly reduced WSS was shown at Ps2 and a maximum WSS was shown at P1. Model 2 showed the biggest change in shear stress and model 3 showed the smallest change in shear stress. In addition, as the stenosis and Reynolds number increased, maximum WSS increased and minimum WSS decreased.

**Figure 6:**
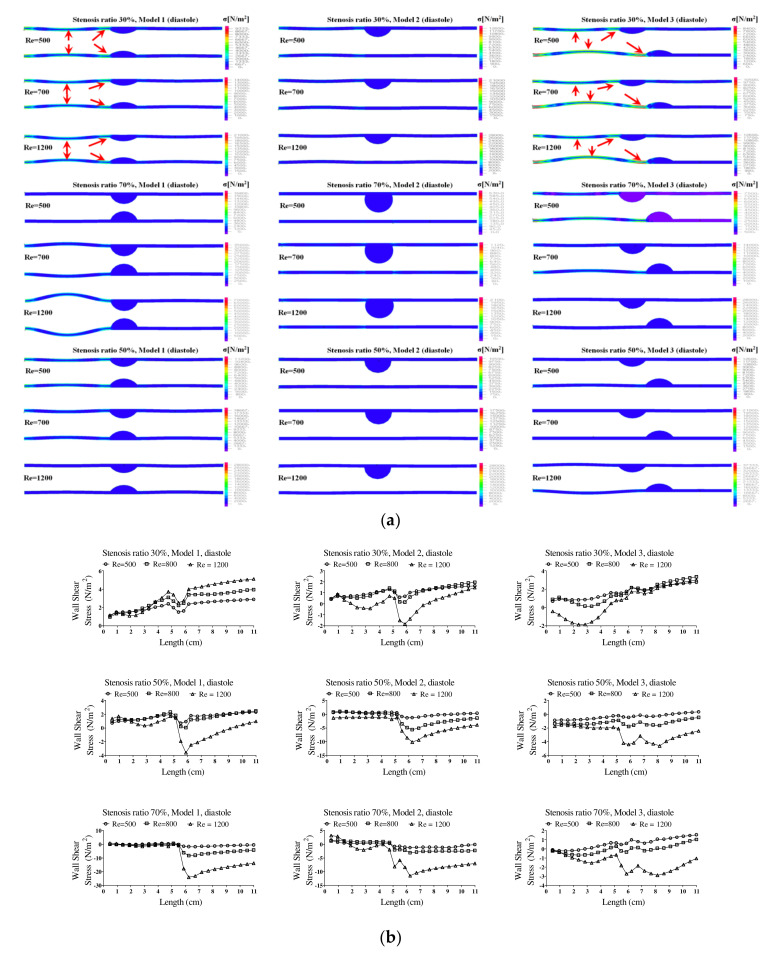
The wall load distribution and wall shear stress on the wall of blood vessels in the pulsatile flow. (**a**) Diastolic wall load distribution, (**b**) diastolic wall shear stress.

Figure 6 shows the wall load distribution and wall shear stress (WSS) on the wall of the blood vessels in the pulsatile flow field based on changes to the stenosis ratio at each point of the modules. In this figure, the WSS implies the hemodynamic force acting on the inner wall of the blood vessel due to the pulsatile flow field in blood vessel. When the stenosis rate was 30% during diastole, the minimum shear stress was shown at P1 in models 1 and 3 and it was shown at Ps2 in model 2. In addition, the minimum shear stress was moving toward the entrance region with an increasing Reynolds number in model 3. When the stenosis rate was 70%, the shear stress was significantly changed in the region after Ps2 with an increasing Reynolds number. The WSS on the wall of the blood vessel during systole showed the highest changes in shear stress in model 1 and the lowest changes in shear stress in model 3. Changes in shear stress were increasing with an increasing Reynolds number. High shear stress was shown in the region ahead of Ps1 and it had a negative value in the region after Ps1. The maximum shear stress was increased with an increasing stenosis rate and Reynolds number, and the minimum shear stress was decreased. P1, in which maximum shear stress was formed, was predicted to be the region where the structure of the wall of the blood vessel was likely to be changed. This region is thought to be the stagnation point of blood, in which platelets, which are a blood parameter, begin to be deposited on an internal wall of the blood vessel and cause the blood clots to form. Platelets, which receive high shear stress, were stagnant at P1 due to the recirculation vortex; they are more likely to be attached to the wall of a blood vessel with increasing time. In these regions, the formation of blood clots is prone to occur, and an aneurysm is frequently formed in regions where blood clots are formed on an internal wall of a blood vessel [4,7]. In all models, points where the maximum shear stress was formed were almost same, but the points where the minimum shear stress was formed were shown differently. Asymmetry of the stenosis shape was found to have a big impact on changes in shear stress.

**Figure 7:**
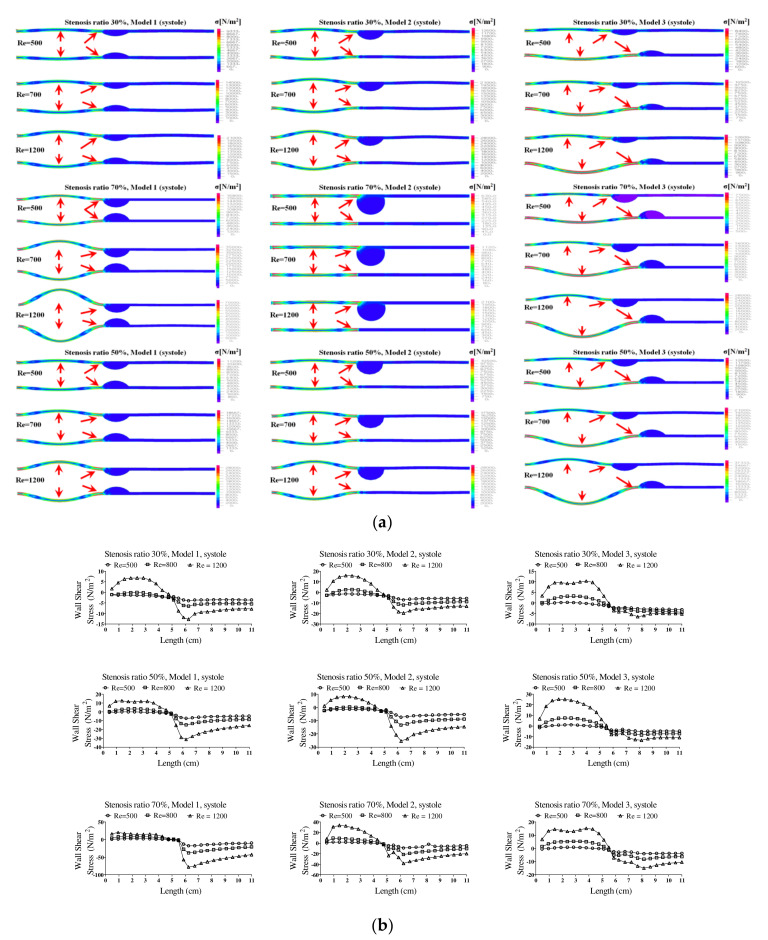
The wall load distribution and wall shear stress on the wall of blood vessels in the pulsatile flow. (**a**) Systolic wall load distribution, (**b**) systolic wall shear stress.

Additionally, Figure 7 shows the wall load distribution on the wall of blood vessels based on changes in the stenosis rate. In models 1 and 2, P1 and Ps1 received more loads. It is thought that the flow of blood at P1 was not smooth due to the stenosis of blood vessels; the diameter of blood vessel was increased and the wall load of blood vessels was increased by movement of the wall load of blood acting on blood vessels. In addition, since the diameter of blood vessels was increased at P1, maximum wall loads and flow energy of blood were concentrated at Ps1. Thus, it was expected that the wall load of blood vessels at Ps1 would show as high. In Model 3, however, maximum wall loads were applied in the region between P1 and Ps1, and maximum wall loads were concentrated at Ps3. In addition, as shown in the results of a continuous flow, maximum wall loads were concentrated at P1 and Ps1 during diastole. However, the maximum wall loads were concentrated at P1, Ps1, Ps3, and the region between P1 and Ps1 during systole. When the results of modeling were reviewed, the wall load acting at P1 and the region between P1 and Ps1 increased the diameter of blood vessels; however, it was expected that it may not have had a significant impact on the destruction of blood vessels, if it was not operated over the limits of elasticity of the blood vessels. Because loads concentrated at point Ps1 and Ps3 were relatively higher than those in other regions, if the destruction of blood vessels occurred, this was the place it was expected to occur. However, because the results of this study were made by a modeling, it is thought that it may be different from actual clinical cases.

Blood flow in the blood vessels in which stenosis occurred showed a lot of differences compared with that in a healthy condition. In addition, it was found that partial turbulence was formed by the vortex occurring in a region before and after the stenosis. Since the inertial force was close to ‘0’ in the stagnation region close to the wall, the main flow caused by a pressure gradient and the fluid flow flowing in an opposite direction were dominant. At the time, flow conflicted with inertial flow in the adverse pressure gradient region of the wall of the blood vessel. Thus, the point occurred in which two flows were offset to ‘0’ [6,17]. According to previous studies, it has been reported that the presence of stenosis in a blood vessel increases the possibility of the formation of turbulence. Additional stress by turbulence and changes in velocity may cause change in the function and structure of the wall of a blood vessel. Formation of turbulence is the region in which it is highly likely to cause the rupture of a blood vessel due to additional stress caused by turbulence [16,18,19].

The pattern of fluid flow was shown in various ways based on the shape of the stenosis with an increasing stenosis rate, and the recirculation region and flow separation region were shown differently. In the figure, the recirculation region and other regions where the flow separation was made are marked with red circles. In addition, the recirculation region was shown as wider with an increasing Reynolds number and stenosis rate. It is known that the occurrence of a vortex related to the recirculation region and stagnation gives more effects on behaviors of suspended solids in a blood vessel and it is closely associated with the movement of platelets, which are the cause of a thrombosis. If the blood stagnates in the region after stenosis of blood caused by an occurrence of a vortex, injury happens in the endothelial cells and sufficient nutrients are not supplied in the wall of the blood vessel. Therefore, due to the characteristics of an unstable flow in the region after the stenosis, it can proceed more quickly [20]. Because such flow separation phenomenon does not have a pressure gradient in a direction perpendicular to the boundary layer in the area of a flow boundary layer, pressure distribution within the boundary layer becomes equal to that of the external non-viscous flow. However, the fluid particle loses its velocity near the surface of the wall of the blood vessel within the boundary layer due to viscous action in the surface of the wall of the blood vessel. Therefore, if the fluid particle close to the wall of the blood vessel reaches a certain region while it flows along the wall of the blood vessel, it does not overcome the adverse pressure gradient and it flows in a direction of the adverse pressure gradient. Flow separation is also made. It is not made in the flow area with a favorable pressure gradient [21].

In addition, hemodynamic characteristics are various based on the morphological characteristics of blood vessels, and the possibility and progress of an aneurysm may vary. If the stenosis is formed in the blood vessels, the smooth flow is disturbed. Thus, the vortex is generated and pressure drop is increased. Both continuous flow and pulsatile flow show the maximum flow rate in the region of stenosis. The recirculation region is formed by the occurrence of a partial vortex in the regions before and after stenosis. The maximum flow velocity and the recirculation region are shown in different locations based on the shape of the stenosis. Recirculation regions are formed more extensively as the stenosis and Reynolds number are increased. When the shape of the stenosis is asymmetric, the generation of a recirculation region and vortex affects behaviors of various suspended solids with an increasing stenosis rate. It is also closely associated with behaviors of platelets, which are the cause of a thrombosis. The pressure drop is increased and the changes in velocity are also shown significantly with an increasing stenosis rate and Reynolds number. When the stenosis rate is the same, the pressure drop and the changes in velocity are bigger in a symmetric structure than those in an asymmetric structure. Changes in the WSS show the maximum value in all regions of a stenosis and the minimum value is shown in point of the stenosis. The minimum shear stress is formed in different locations based on the stenosis shape model. It is thought that the regions with high shear stress may destroy or damage blood endothelial cells and the regions with low shear stress may cause damage to the blood vessels by increasing the stagnation time of blood flow. The maximum WSS is increased and the minimum WSS is decreased with an increasing stenosis rate and Reynolds number. We suggest that pressure distribution across the stenosis is more important for atherosclerotic material (plaque) vulnerability. There is a pressure drop across the atherosclerotic material because of the stenosis. According to the Bernoulli principle, this increased blood velocity produces a lower lateral blood pressure acting on the atherosclerotic material. Thus, a pressure gradient buildup is created across the atherosclerotic material that could rupture it. Any increase in systemic pressure or increase in the narrowing of the lumen would further increase the velocity through the narrowed lumen and increase the pressure drop [22,23].

Atherosclerotic material stress may be a more important factor when the mechanism of atherosclerotic material rupture is considered [12,22,23,24,25]. The arterial wall continuously interacts with hemodynamic forces, which include WSS and blood pressure. Atherosclerotic material stress is the result of external hemodynamic forces. Atherosclerotic material rupture itself represents structural failure of a component of the diseased vessel, and it is, therefore, reasonable to propose that the biomechanical properties of atheromatous lesions may influence their vulnerability to rupture [11,26].

Fluid velocity, atherosclerotic material deformation, and atherosclerotic material internal stress were calculated. A stress of 300 KPa was used as the threshold to indicate high risk of atherosclerotic material rupture. After data analysis, Li et al. concluded that there is a direct correlation between the degree of stenosis and the thickness of the fibrous cap [27]. It is common practice for a physician to perform surgery for a stenosis greater than 70% due to high rupture risks [13,28]. Li et al. [23] showed in the results that there is still a high risk for rupture of a stenosis between 30% and 70% depending on the thickness of the fibrous cap. The critical thickness for this range of stenosis was shown to be 0.5 mm. Because the occurrence of a stenosis in blood vessels may affect the elastic behavior of the wall of a blood vessel, it is expected that the pressure is increased due to an unstable blood flow and the aneurysm, which is the phenomenon of a swelling blood vessel, occurs in all regions of a stenosis.

## 3. Materials and Methods

### 3.1. Numerical Model

**Figure 8:**
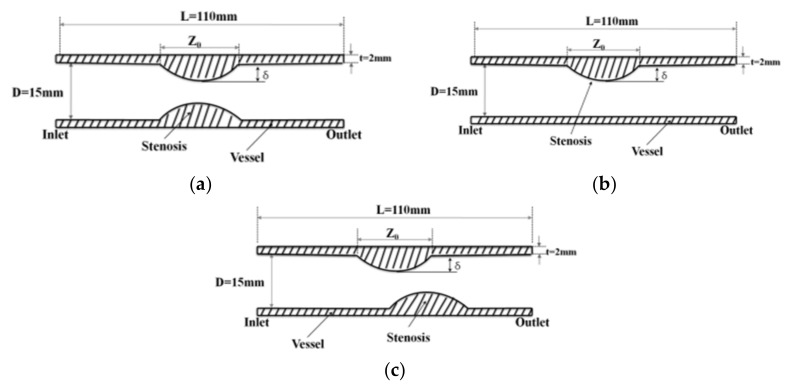
Geometry of the stenosed blood vessel. (**a**) Model 1, (**b**) model 2, and (**c**) model 3. δ = depth of stenosis.

**Figure 9:**
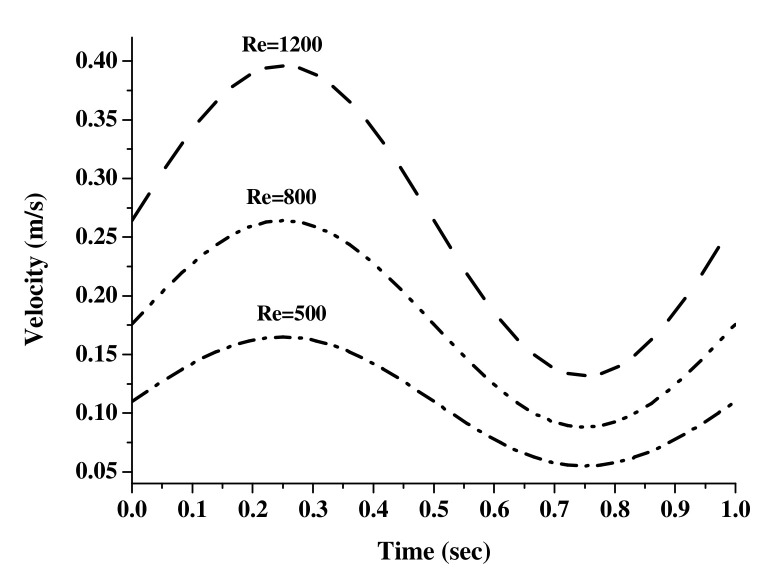
The pulsatile velocity profile at the inlet with the change of Reynolds numbers.

**Figure 10:**
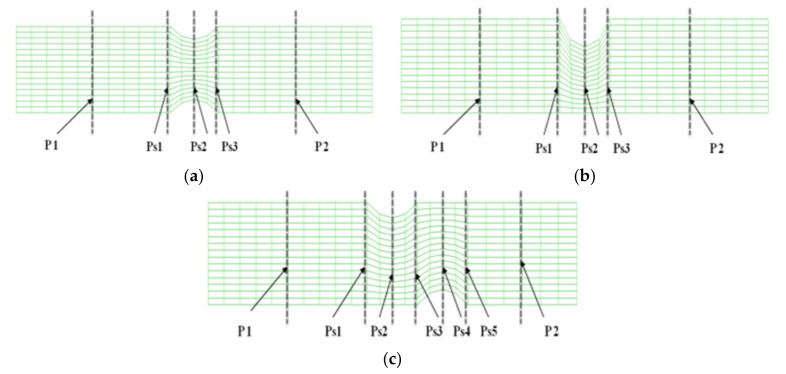
Grid mesh used in the fluid and solid models. (**a**) Model 1, (**b**) model 2, and (**c**) model 3.

There is much focus on attempting to numerically understand the mechanics of AAA rupture; however, only limited work has focused on the development of experimental methods of determining rupture potential. Morris et al. [29] reported the use of the photoelastic method in determining wall stress distributions in an idealized AAA case that used dimensions taken from a comprehensive study. Previous work by Doyle et al. identified the sites of rupture in idealized AAA models by performing in vitro rupture studies and comparing these findings with those predicted using numerical modeling (Figure 8). Mechanical characterization of the material used was also performed, along with the examination of silicone model wall thickness [30] (Figure 9 and Figure 10).

A human abdominal aorta with combined stenosis and aneurysm was collected after surgical removing. A computational fluid dynamics model was constructed from two-dimensional (2D) rational angiography images, a pulsatile flow calculation was performed, and hemodynamic characteristics were analyzed. It was applied on the blood flow in abdominal aortic blood vessels in which stenosis occurred by using the commercial finite element software ADINA Ver 8.5 (ADINA R & D, Inc., Lebanon, MA, USA) on fluid-solid interactions. The geometry of a stenosed artery blood vessel shown in Figure 8 was used in order to analyze the blood flow of abdominal aortic blood vessels in which stenosis occurred. Model 1 was used to establish modeling for the structure of a symmetric stenosis. Model 2 was used to establish modeling for the structure of a stenosis occurring on one side. Model 3 was used to establish modeling for the structure of a nonsymmetric stenosis. The diameter (D) of abdominal aortic blood vessels was 15 mm and length (L) was 110 mm. The length (Z_0_) of the part where the stenosis occurred was 15 mm and the thickness of the vessel wall was 2 mm. Because the shapes of the stenoses were very different, there was no stylized shape. Therefore, the geometric shape of a stenosed section in a numerical model was assumed to be a cosine function and Equation (1) was used. The variables to represent the size of a stenosed section were defined as Equation (2) for a stenosis ratio [22]. The stenosis ratios of 20, 50, and 70% were set depending on the extent of decrease of a cross-sectional area of a blood vessel in a stenosed section in a numerical model.

(1) $$\frac{r\left(z\right)}{R}=\left\{\begin{array}{ll} 1-\frac{\delta }{2}\left[1+\cos\left(\frac{\pi z}{z_{0}}\right)\right] & \;\mathrm{if}\;\left|z\right|\leq z_{0}\\ 1 & \;\mathrm{if}\;\left|z\right|\geq z_{0}\; \end{array}\right.$$

(2) $$s=\left(d-\delta \right)/d\times 100\%$$

In this study, it was a lattice generation model according to the shape of the stenosis. In Models 1 and 2, P1 was the central point of the pre-stenosis, Ps1 was the starting point of the stenosis, Ps2 was the stenosis, Ps3 was the end of the stenosis, and P2 was the central point of the post-stenosis. In model 3, Ps3 denoted the first stenosis endpoint, Ps4 denoted the second stenosis, and Ps5 denoted the second stenosis endpoint. In the case of the total elements of the generated grid, the structural model had 96 pieces and the fluid model had 360 pieces.

The wall was fixed by constraining the degrees of freedom of the nodes located at the inlet and outlet rings. The solution was obtained using an element and node mesh with a time step of 0.4 ms, and five nonlinear iterations per time step, for a total of three cardiac cycles.

### 3.2. Boundary Condition

It was assumed that the blood vessel wall was the elastic wall with a constant thickness. The density of the vessel wall was 2000 [kg/m^3^], Young’s modulus was 0.7 × 10^6^ [N/m^2^], and the Poisson ratio was 0.49. Blood used in this study was assumed as a uniform, incompressible, and isothermal Newtonian fluid; the density was 1060 [kg/m^3^] and dynamic viscosity was 0.0035 [kg/m·s], respectively [16]. Blood behaves as a non-Newtonian fluid at shear rates above 100 s^−1^ [26,31,32] Reynolds numbers. The vessel was assumed to be fixed and the degree of freedom was a fixed variable. The paper describes the calculation of an effective Newtonian viscosity, which captured the non-Newtonian effects for this flow situation [33]. Sud and Sekhon [34] presented a mathematical model for flow in single arteries subject to a pulsatile pressure gradient as well as the body acceleration. In their analytical treatment, blood was assumed to be a Newtonian fluid and flow as laminar and one dimensional; and the tube wall was assumed to be rigid and uniformly circular [34,35,36]. A fully developed flow was given as an inlet condition to analyze the characteristic of the blood flow within the blood vessels in which the stenosis occurred; numerical analysis with the velocity distribution of the Reynolds numbers (Re) of 500, 800, and 1200 at the inlet was carried out. In addition, the continuous flow and the pulsatile flow, which had the regular shape in the form of a sine function, were given as an inlet condition. If the flow was the pulsatile flow as an inlet condition, it was assumed that the velocity distribution changed with the sine wave form without the occurrence of the reflux of 1 Hz. The initial velocity based on Re was calculated using Equation (3).

(3) $$U\left(t\right)=\bar{U}\left[1+0.5\sin(2\pi t\right)]$$

Figure 9 shows the velocity distribution depending on time used in inlet condition at the pulsatile flow. The velocity based on time had the maximal value at t = 0.25 s and the minimal value at t = 0.75 s, respectively. In the case of the pulsatile flow, the blood vessel wall was assumed as an elastic wall and systole and diastole were repeated depending on the fluid flow. No-slip condition was used in the blood vessel wall.

In this study, the iteration method was set to repeat it up to 15 times per step by using a Newton method, and then it was calculated. A direct computing method with good convergence was used as the method to interpret the fluid-structure interaction (FSI) model. The FCBI (flow condition-based interpolation) element was used for fluid element formulation.

Figure 10 shows the grid generation model based on a structure in which stenosis occurs. In Models 1 and 2, P1 represented the center point of the region before the stenosis. Ps1 represented the start point of the stenosis. Ps2 represented the center point of the stenosis. Ps3 represented the endpoint of the stenosis. P2 represented the center point of the area after the stenosis. In Model 3, P1 represented the center point of the area before the stenosis. Ps1 represented the start point of the first stenosis. Ps2 represented the center point of the first stenosis. Ps3 represented the endpoint of the first stenosis. Ps4 represented the center point of the second stenosis. Ps5 represented the end point of the second stenosis. In the case of total elements of the generated grid, there were 96 structural models and 360 fluid models.

## 4. Conclusions

The purposes of this study were to analyze the stenosis rate in blood vessels, changes in pressure in the blood vessels, and load distribution applied to the blood vessels, and then to examine and predict the effects on the development of an aneurysm and vascular rupture by using computational fluid dynamics (CFD) methods.

As a result of the modeling, when the intravascular stenosis occurred, the smooth blood circulation interfered to generate a vortex and increase a pressure drop. With an increasing stenosis rate and Reynolds number, the pressure drop was increased and the velocity was greatly changed. When the stenosis rate was the same, the pressure drop and the velocity change were larger in the stenosis with a symmetric structure than in the stenosis with an asymmetric one. Maximal changes in wall shear stress were observed in the area before the stenosis and minimal changes were shown in the stenosis areas. The minimal shear stress occurred at different locations depending on the stenosis shape models. The area with high shear stress destroyed or damaged the endothelial cells, and the area with low shear stress increased the stagnation time of blood flow and caused vascular injury. With an increasing stenosis rate and Reynolds number, the maximal wall shear stress was increased and the minimal wall shear stress was decreased. As the stenosis progressed, the possibility of stenosis growth increased and arteriosclerosis developed quickly.

Because the occurrence of vascular stenosis affected the elastic behavior of the vessel wall, the unstable blood flow in the area before the stenosis was expected to result in an increase in pressure and the occurrence of the aneurysm, which was the phenomenon of the swelling of the blood vessel. As the stenosis progressed, the diameter of the blood vessels was increased in the area before the stenosis, and the thickness of the vessel wall and the shape of the stenosis were predicted to have significant effects on the changes in the diameter of blood vessels.

In this study, flow separation occurred in Ps3, the part where the stenosis ended; when the stenosis rate was 70%, flow separation also occurred in Ps3 and P2. In the flow separation phenomenon, there was no pressure gradient in the direction perpendicular to the boundary layer in the flow boundary layer region, so the pressure distribution in the boundary layer was the same as the pressure distribution in the external non-viscous flow. However, the fluid particles near the surface of the object in the boundary layer lost speed due to the viscous action that must satisfy the adhesion condition on the surface of the object and, thus, did not have enough dynamic pressure to achieve equilibrium with the pressure. Therefore, the fluid particles near the wall flowed along the wall and, when they reached a certain point, they could not overcome the reverse pressure gradient and flowed in the reverse pressure gradient direction, causing flow separation to occur. Separation did not occur in the flow region with a net pressure gradient. However, so far, all the experimental models were based on the boundary layer region. It is necessary to compare with an outlet boundary condition to further validate our results in the future.

Through this study, it is thought that characteristics of blood flow in the abdominal aorta where a stenosis is formed will be helpful in understanding the mechanism of growth of atherosclerosis and the occurrence and rupture of abdominal aortic flow.

Additionally, it is judged that it is necessary to conduct an experimental study based on this study and compare it with the simulation results.

## Data Availability

The data presented in this study are available in this article.

## Sample Availability

Samples of the compounds are not available from the authors.

## Nomenclature

AAA	abdominal aortic aneurysm
AS	aortic stenosis
D	diameter
CFD	computational fluid dynamics
FSI	Fluid-structure interaction
L	length
LDL	low-density lipoprotein
P1	the center point of the region before a stenosis
P2	the center point of the area after a stenosis
Ps1	the start point of a stenosis
Ps2	the center point of a stenosis
Ps3	the endpoint of a stenosis
Ps3	the endpoint of the first stenosis
Ps4	the center point of the second stenosis
Ps5	the endpoint of the second stenosis
Re	Reynolds number
